# Cellular abundance-based prognostic model associated with deregulated gene expression of leukemic stem cells in acute myeloid leukemia

**DOI:** 10.3389/fcell.2024.1345660

**Published:** 2024-03-07

**Authors:** Dong-Jin Han, Sunmin Kim, Seo-Young Lee, Su Jung Kang, Youngbeen Moon, Hoon Seok Kim, Myungshin Kim, Tae-Min Kim

**Affiliations:** ^1^ Department of Medical Informatics, College of Medicine, The Catholic University of Korea, Seoul, Republic of Korea; ^2^ Cancer Research Institute, College of Medicine, The Catholic University of Korea, Seoul, Republic of Korea; ^3^ Department of Biomedicine & Health Sciences, Graduate School, The Catholic University of Korea, Seoul, Republic of Korea; ^4^ Department of Laboratory Medicine, College of Medicine, The Catholic University of Korea, Seoul, Republic of Korea; ^5^ Catholic Genetic Laboratory Center, Seoul St. Mary’s Hospital, College of Medicine, The Catholic University of Korea, Seoul, Republic of Korea; ^6^ CMC Institute for Basic Medical Science, The Catholic Medical Center of The Catholic University of Korea, Seoul, Republic of Korea

**Keywords:** leukemic stem cell, cellular deconvolution, acute myeloid leukemia, prognostic model, transcriptomic profiles

## Abstract

**Background:** Previous studies have reported that genes highly expressed in leukemic stem cells (LSC) may dictate the survival probability of patients and expression-based cellular deconvolution may be informative in forecasting prognosis. However, whether the prognosis of acute myeloid leukemia (AML) can be predicted using gene expression and deconvoluted cellular abundances is debatable.

**Methods:** Nine different cell-type abundances of a training set composed of the AML samples of 422 patients, were used to build a model for predicting prognosis by least absolute shrinkage and selection operator Cox regression. This model was validated in two different validation sets, TCGA-LAML and Beat AML (*n* = 179 and 451, respectively).

**Results:** We introduce a new prognosis predicting model for AML called the LSC activity (LSCA) score, which incorporates the abundance of 5 cell types, granulocyte-monocyte progenitors, common myeloid progenitors, CD45RA + cells, megakaryocyte-erythrocyte progenitors, and multipotent progenitors. Overall survival probabilities between the high and low LSCA score groups were significantly different in TCGA-LAML and Beat AML cohorts (log-rank *p*-value = $3.3\times 10^{-4}$ and $4.3\times 10^{-3}$ , respectively). Also, multivariate Cox regression analysis on these two validation sets shows that LSCA score is independent prognostic factor when considering age, sex, and cytogenetic risk (hazard ratio, HR = 2.17; 95% CI 1.40–3.34; *p* < 0.001 and HR = 1.20; 95% CI 1.02–1.43; *p* < 0.03, respectively). The performance of the LSCA score was comparable to other prognostic models, LSC17, APS, and CTC scores, as indicated by the area under the curve. Gene set variation analysis with six LSC-related functional gene sets indicated that high and low LSCA scores are associated with upregulated and downregulated genes in LSCs.

**Conclusion:** We have developed a new prognosis prediction scoring system for AML patients, the LSCA score, which uses deconvoluted cell-type abundance only.

## Introduction

It has long been recognized that hematopoietic defects underlie the pathogenesis of acute myeloid leukemia (AML) (Yamashita et al., 2020). Several decades ago, leukemic stem cells (LSCs) were proposed to be a major cause of leukemia (Vetrie et al., 2020), and also associated with drug resistance and disease relapse (Zhai and Jiang, 2022). However, less is known about the cellular origins of LSCs, and the abnormal bone marrow microenvironment that can facilitate their survival (Chen et al., 2022).

Transcriptomic profile-based disease subclassification has been applied to various types of cancer, including hematopoietic malignancies of AML and myelodysplastic syndrome (Shiozawa et al., 2017; Cheng et al., 2022). These studies demonstrate that patients can be segregated into subgroups by gene expression profiles alone with potential clinical utility; however, the bulk-level molecular taxonomy hardly probes the direct clinical-genetic association (e.g., LSCs and prognosis) largely due to limited resolution and tumor heterogeneity.

To overcome tumor heterogeneity along with elevated cellular resolution, single cell RNA sequencing (scRNA-seq) has emerged to facilitate transcriptomic profiling at single-cell resolution (Wang et al., 2022; Zhai et al., 2022). Although the transcriptional profiling of individual cells by scRNA-seq has revealed unique cellular populations and dysregulated cellular ecosystems (Lu et al., 2022), the current availability of scRNA-seq in hematologic malignancies for large-scale clinical correlation analyses is still limited.

The use of deconvolution-based algorithms has been used as an approach to address tumor heterogeneity. The deconvolution of estimated cell type fractions from bulk RNA-seq data can be achieved with or without the prior information of cell type-specific expression or signatures. The latter has a particular advantage, such as the discovery of latent features (i.e., novel cell types) (Miller et al., 2022), but more commonly used regression-based methods, such as CIBERSORT (Newman et al., 2015) rely on the former approach. The signature matrix represents a subset of genes with cell type-specific expression. The biological insights (e.g., cell types to be deconvoluted) and also the performance of signature-based deconvolution are dependent upon the signature matrices (Newman et al., 2015; Wang et al., 2019; Li et al., 2020; Chu et al., 2022). Signature matrices have often been designed in the process of devising their accompanying deconvolution algorithms, e.g., LM22 of CIBERSORT and the signature matrix composed of 13 cell types for validation of LinDeconSeq (Li et al., 2020).

There have been efforts to design gene signatures to represent the hematologic hierarchy and a study demonstrates that the deconvoluted cellular fractions can be implemented into a prognostic scoring system, for instance, GES25-150 was used to calculate CTC score (Dai et al., 2021). They used scRNA-seq data composed of 21 cell types derived from bone marrow specimens of 16 AML patients (van Galen et al., 2019) highlighting a deconvolution-based prognostic system based on the bulk-level transcriptome data. Although other studies have proposed scores based on gene expression that can be used to predict the prognosis of patients (Gentles et al., 2010; Ng et al., 2016; Docking et al., 2021), these clinic-oriented score systems do not take into account the direct relationship between LSCs and patients’ prognosis.

In this study, we built a signature matrix representing the 9 cell types encompassing both normal progenitors and LSC lineages (HemLin9). The deconvoluted cellular fractions of 9 cell types of the AML cohort composed of 422 patients [GSE37642 (GPL96) (Kuett et al., 2015)] were subject to least absolute shrinkage and selection operator (LASSO) for feature selection. Using the selected features, we formulated the leukemic stem cell activity (LSCA) score that stratifies the patients with respect to clinical outcomes. Then, two different cohorts were further used to validate the LSCA score. In addition, we performed gene set variation analysis (GSVA) using six LSC-related gene sets to show that the LSCA score is associated with LSC functionality, that is, gene sets that are up or downregulated transcriptionally in LSCs are more enriched in patients with a high or low LSCA score, respectively. Lastly, we found that this tendency is shown not only in bulk-level expression profiles but also in cell type-specific expression data inferred by CIBERSORTx high-resolution mode.

## Materials and methods

### HemLin9 signature matrix

Differentially expressed genes (DEGs) in cell type sorted microarray data (GSE24006) composed of 9 cell types were obtained through the “lmFit” function of the limma (Ritchie et al., 2015) R package. The cell types were as follows: Lin-CD34^−^, AML blast; common myeloid progenitors (CMPs, Lin-CD34^+^CD38^+^CD123+CD45RA-); granulocyte-monocyte progenitors (GMPs, Lin-CD34^+^CD38^+^CD123+CD45RA+); hematopoietic stem cells (HSCs, Lin-CD34^+^CD38^−^CD90^+^CD45RA-); leukemic progenitor cells (LPCs, Lin-CD34^+^CD38^+^); LSC, Lin-CD34^+^CD38^−^CD90^−^; megakaryocyte-erythrocyte progenitors (MEPs, Lin-CD34^+^CD38^+^CD123-CD45RA-); multipotent progenitors (MPPs, Lin-CD34^+^CD38^−^CD90^−^CD45RA-); CD45RA + cells (RApos, Lin-CD34^+^CD38^−^CD90^−^CD45RA+). The DEGs criteria were logFC (fold change) ≥ 1 and adj.P.Val ≤0.05 except LSCs, RApos, and MPPs. To find at least 25 DEGs for each cell type, we made an exception by setting adj.P.Val for LSC, RApos, and MPP at 0.1, 0.1, and 0.2, respectively. We sorted DEGs in the order of logFC and then obtained 150 DEGs at most by each cell type. Five cell types (AML_blast, CMP, GMP, MEP, and MPP) have 150 DEGs and the other 4 cell types, HSC, LPC, LSC, and RApos have 72, 28, 56, and 25 DEGs (Supplementary Figure S1E). The full list of 841 non-redundant DEGs is available in Supplementary Table S1. We further tested four different signature matrices by using the top 25, 50, 100, and 150 DEGs based on logFC and the resulting matrices (composed of non-redundant 205, 361, 609, and 841 genes, respectively) can be found in Supplementary Tables S2–S5. Heat maps corresponding to individual signature matrices are represented in Supplementary Figures S1A–D.

### Preparing training and validation data sets

The training set (GSE24006) was downloaded from the GEO database through the GEOquery (Davis and Meltzer, 2007) R package. Since a gene symbol can correspond to multiple probe IDs, we calculated the median expression value of probes having the same gene symbol. We downloaded TCGA-LAML RNA-seq data composed of 179 patients through https://gdc.cancer.gov/about-data/publications/laml_2012 (RNAseq GAF 2.0 normalized reads per kilobase of transcript per million mapped reads, RPKM). Also, the clinical data of TCGA-LAML was downloaded from the same web page (Patient Clinical Data) except for overall survival time and vital status in TCGA pan-cancer clinical data https://gdc.cancer.gov/about-data/publications/pancanatlas (TCGA-Clinical Data Resource Outcome). Beat AML RNA-seq data and clinical data were acquired from the Supplementary Data (Supplementary Tables S5, S8, respectively) of Tyner et al. (2018). Clinical information such as age, sex, French-American-British (FAB) classification, and cytogenetic risk of the three cohorts are presented in Table 1.

**TABLE 1:** Clinical information on the three cohorts. One training set, GSE37642, and two validation sets, TCGA-LAML and Beat AML, were used for creating and validating a LASSO Cox regression model. Age, sex, FAB classification, and cytogenetic risk of patients are described in the table. Sex and cytogenetic risk are not available in GSE37642.

	GSE37642	TCGA-LAML	Beat AML
**N**	422	179	451
Sample source	Bone marrow (Mononuclear cells)	Bone marrow	Bone marrow (239)
			Leukapheresis (9)
			Peripheral blood (203)
Age			
Range (Median)	18–83 (57)	18–88 (58)	2–87 (61)
Sex			
Female	—	84	193
Male	—	95	258
FAB classification			
M0	14	16	6
M1	84	42	8
M2	117	41	10
M3/M3v	19	16	10
M4/M4Eo	104	36	25
M5/M5a/M5b	47	21	32
M6	15	2	0
M7	2	3	2
Unknown/NOS	20	2	358
Cytogenetic risk			
Favorable	—	33	131
Intermediate	—	104	150
Poor	—	40	169
NA	—	2	1

FAB, French-American-British; NOS, not otherwise specified; NA, not available.

### Pseudo-bulk gene expression data design and deconvolution tool candidates

There are tools for inferring cell-type abundance such as CIBERSORTx (Newman et al., 2019), LinDeconSeq (Li et al., 2020), MuSic (Wang et al., 2019), and BayesPrism (Chu et al., 2022). Among these, scRNA-seq data are required to use MuSic and BayesPrism. Instead, we used a deconvolution tool FARDEEP (Hao et al., 2019) along with CIBERSORTx and LinDeconSeq for the deconvolution performance comparison. We created pseudo-bulk gene expression data with cell-type sorted microarray data (GSE24006). First, we calculated medians of the same cell type by gene. Then, we made a random cell-type fraction matrix composed of 100 samples. After that, we multiplied random fractions and median expression values by gene. Finally, pseudo-bulk expression data was made by summing gene expression values of the 9 cell types by gene.

### Deconvolution of cell type-specific abundance

We used the CIBERSORTx (Newman et al., 2019) “Impute Cell Fractions” module to calculate the cell type compositions of each patient. For validation, RPKM gene expression data of TCGA-LAML and Beat AML were used respectively. We disabled quantile normalization as recommended. Also, we did not apply the “Enable batch correction” and “Run in absolute mode” options. All options were default. All results in this study were obtained by using HemLin9 signature matrix composed of 50 DEGs, unless otherwise stated.

### LASSO Cox regression

R package glmnet (Simon et al., 2011) (v4.1.7) was utilized for conducting LASSO Cox regression to identify the impact of cell type abundances on the prognosis of AML patients. Among 422 patients in the GSE24006 dataset, overall survival data was only available for 417. Therefore, we used these samples as a training set. Cell type fraction values of these 417 samples were used for an input matrix, and overall survival time and vital status were used for the response variable. We performed 10-fold cross-validation using the “cv.glmnet” function and selected the lambda value that resulted in the minimum error. Then, we built a LASSO Cox regression model using the “glmnet” function. We iterated this procedure 100 times, filtering out cell types with zero coefficients occurring more than five times. Finally, we considered the mean values of the 100 coefficients as the final coefficient of the cell type. Among four signature matrices with varying gene sizes, that composed of 50 DEGs showed the best performance based on hazard ratio (HR) and its *p*-value along with survival log-rank test *p*-value (Supplementary Figure S4). Therefore, we selected this model and the corresponding signature matrix as a reference and applied them to validation sets. A patient-specific score can be calculated by summing the product of each cell type fraction of the patient and the cell type coefficient. We called this the LSCA score. The LSCA score can be expressed as the following. $\text{LSCA score}=-2.15\times F_{\text{GMP}}\;-\;1.64\times F_{\text{CMP}}+0.37\times F_{\text{RApos}}+0.49\times F_{\text{MEP}}+4.52\times F_{\text{MPP}}$ If you want to check the scripts for model construction and validation, please visit the following website: www.github.com/LabTMK/LSCA.

### Multivariate Cox regression

Multivariate Cox regression analysis was performed by the “coxph” function of survival (Therneau and Grambsch, 2000) (v3.5.5) and the “forest_model” function of forestmodel (Kennedy, 2020) (v0.6.2) R packages. In calculating hazard ratios of TCGA-LAML and Beat AML data sets, four variables were included: age, sex, cytogenetic risk, and LSCA score. Since cytogenetic risk and sex are not available in training set, GSE37642, we instead incorporated the mutation and fusion in *RUNX1* gene as covariables. To concord with TCGA-LAML data set, we renamed terms of cytogenetic risk “FavorableOrIntermediate,” “IntermediateOrAdverse,” and “Adverse” in Beat AML data set as “Favorable,” “Intermediate,” and “Poor,” respectively.

### Gene set variation analysis

GSVA (Hanzelmann et al., 2013) (v 1.42.0) was performed separately on three transcriptomic data. Using the msigdb (Bhuva et al., 2021) (v1.2.0) R package, we manually inspected and selected six gene sets related to LSCs within C2 chemical and genetic perturbations (CGP); GENTLES_LEUKEMIC_STEM_CELL_DN, GENTLES_LEUKEMIC_STEM_CELL_UP, EPPERT_LSC_R, EPPERT_CE_HSC_LSC, GAL_LEUKEMIC_STEM_CELL_DN, and GAL_LEUKEMIC_STEM_CELL_UP. We used the “gsva” function setting kcdf option as “Gaussian” and other options as default. After calculating the enrichment score (ES) of each patient in each gene set, we calculated the Pearson correlation coefficients (PCCs) among gene sets, LSCA, and CTC score using corrplot (Wei and Simko, 2021) (v0.92).

CIBERSORTx docker was downloaded from https://cibersortx.stanford.edu/, and high-resolution analysis was performed to acquire nine sets of cell type-specific gene expression data using the docker. Each of the training and validation data sets was used as bulk gene expression data, and HemLin9 composed of the top 50 DEGs was used as a signature matrix. Quantile normalization was disabled, and all other options were set to default. The ES of the six gene sets by each patient was calculated in not only bulk but also cell type-specific gene expression data. Then, patients were divided into high and low LSCA groups by the median score of each data set. Finally, the log2-scale fold change was calculated between high and low LSCA groups using the limma R package. Gene sets with no valid genes were ignored and shown as gray in the heatmap.

## Results

### Signature matrix (HemLin9) of hematologic lineages

The signature matrices were built by cell type-specific DEGs (Supplementary Tanle S1). A total of 841 non-redundant genes are identified as the DEGs excluding 89 genes shared by more than 1 cell type. Based on a metric of differential expression (logFC), we made four different signature matrices with a varying number of genes (i.e., top 25, 50, 100, and 150 DEGs; Supplementary Tables S2–S5). From these, the top 50 gene-based signatures demonstrated a superior performance and were selected (Supplementary Figure S4) (HemLin9 afterward). The number of genes representing individual cell types is shown (Supplementary Figure S1E). The cell type-specific gene expression of these signature matrices is shown by row-wise normalized expression levels (Supplementary Figures S1A–D). Among 72 HSC DEGS, 43 are shared by MPPs, and CMPs share common DEGs with GMPs and MEPs (Supplementary Figures S1F, G).

### Cell-type abundance deconvolution tool selection

Using three different tools, CIBERSORTx, LinDeconSeq, and FARDEEP, the cell-type abundance of 100 samples of pseudo-bulk expression data were deconvoluted using HemLin9 as a signature matrix (see Methods). PCCs between the known cell type fractions and the inferred values, were calculated and represented by bar plots (Supplementary Figure S2). CIBERSORTx and LinDeconSeq showed a comparable performance; however, FARDEEP showed the poorest performance. For subsequent analyses, we selected CIBERSORTx as a cell-type abundance deconvolution tool.

### Cellular abundances of three AML cohorts

**FIGURE 1:**
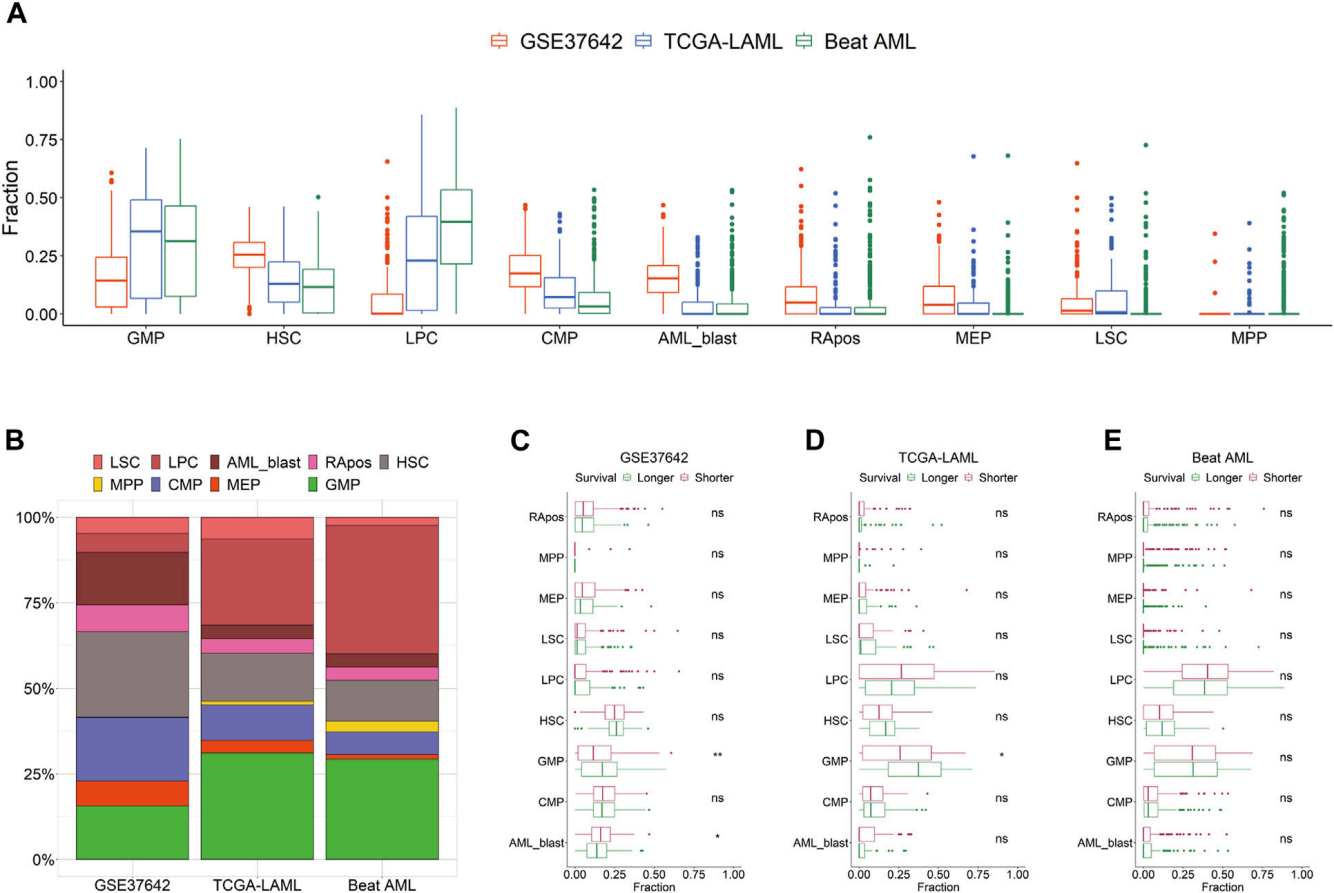
Distribution of 9 cell types by cohort. **(A)** Cell type abundances of GSE37642, TCGA-LAML, and Beat AML are depicted as box plots by cell type. **(B)** Compositions of 9 cell types are shown as a stacked bar chart by cohort. Patients are divided by survival period median into longer or shorter groups in GSE37642 **(C)**, TCGA-LAML **(D)**, and Beat AML **(E)**. Cellular abundances are compared between the two groups in each data set by cell type. Wilcoxon rank-sum test results are shown as ns (non-significant) and asterisks (* and ** indicate *p*-value less than or equal to 0.05 and 0.01, respectively).

We curated the public transcriptomic data of three AML cohorts; the clinical information is summarized in Table 1. Using HemLin9 as a signature matrix, we inferred the cell-type abundances of the three data sets using CIBERSORTx. The cell type compositions of the three data sets were illustrated using box plots for each cell type (Figure 1A), and the abundance distribution of each cohort is depicted in a stacked bar plot (Figure 1B). In signatures, differentiated lineages such as B, T, and NK cells were excluded to focus on 9 cell types that included hematopoietic stem or progenitor cell types. Although a difference in overall cellular abundance was noted across cohorts, the composition of normal progenitors such as MPP, CMP, MEP, and GMP were similar to each other. Given that the heterogeneity of cell type compositions can be observed even within a single cohort (Fan et al., 2023), it is expected that there are differences in cell type compositions among the three cohorts. The cell-type abundance of each patient is sorted by cohort in Supplementary Tables S6–S8.

### Association between survival span and cellular abundances

We hypothesized that the prognosis of patients is impacted by the activity or the abundance of specific cell types. First, we divided patients into two groups, longer or shorter survival, and then determined, which cell type in the three data sets showed a significant difference in abundance between the two groups (Figures 1C–E). All 9 cell types showed no difference in abundance between the two groups in Beat AML. In TCGA-LAML and GSE37642, the abundance of GMP was the only significant difference between the two survival groups. In the CTC score, patients show a good prognosis with higher GMP-like fractions because this cell type has the largest negative coefficient. Also, in the two subgroups of TCGA-AML divided by LinDeconSeq, the prognosis of the GMP fractions high subgroup was better than the other subgroup (Li et al., 2020). Second, we divided patients into two groups, high and low based on the abundance of each cell type. To divide patients into high and low groups, we used the mean values of each cell-type abundance rather than medians to minimize cases that have a zero cutoff value. Among the 9 cell types, significant differences in survival were consistently observed across three AML cohorts between RApos high and low patients (Supplementary Table S9; Supplementary Figures S3A–C). However, when incorporating other clinical features into a multivariate Cox regression, the significance of RApos abundance diminished (Supplementary Figures S3D–F). This indicates that relying on a single cellular feature is insufficient for determining the clinical relevance.

### Modeling of LSCA scores

To assess the potential independent impact of multiple cell type fractions on prognosis, we conducted LASSO Cox regression analysis on the data of 422 AML patients from GSE37642 (GPL96) (Kuett et al., 2015). To ensure robustness, we estimated the coefficients of individual cell types using bootstrapping while filtering out cell types with insignificant values (*p* > 0.05) (see Methods). In the regression, the observed coefficients of GMPs, CMPs, RApos, MEPs, and MPPs (−2.15, −1.64, 0.37, 0.49, and 4.52, respectively) retained the significance and were incorporated into the equation for the prognosis predicting scoring system.

**FIGURE 2:**
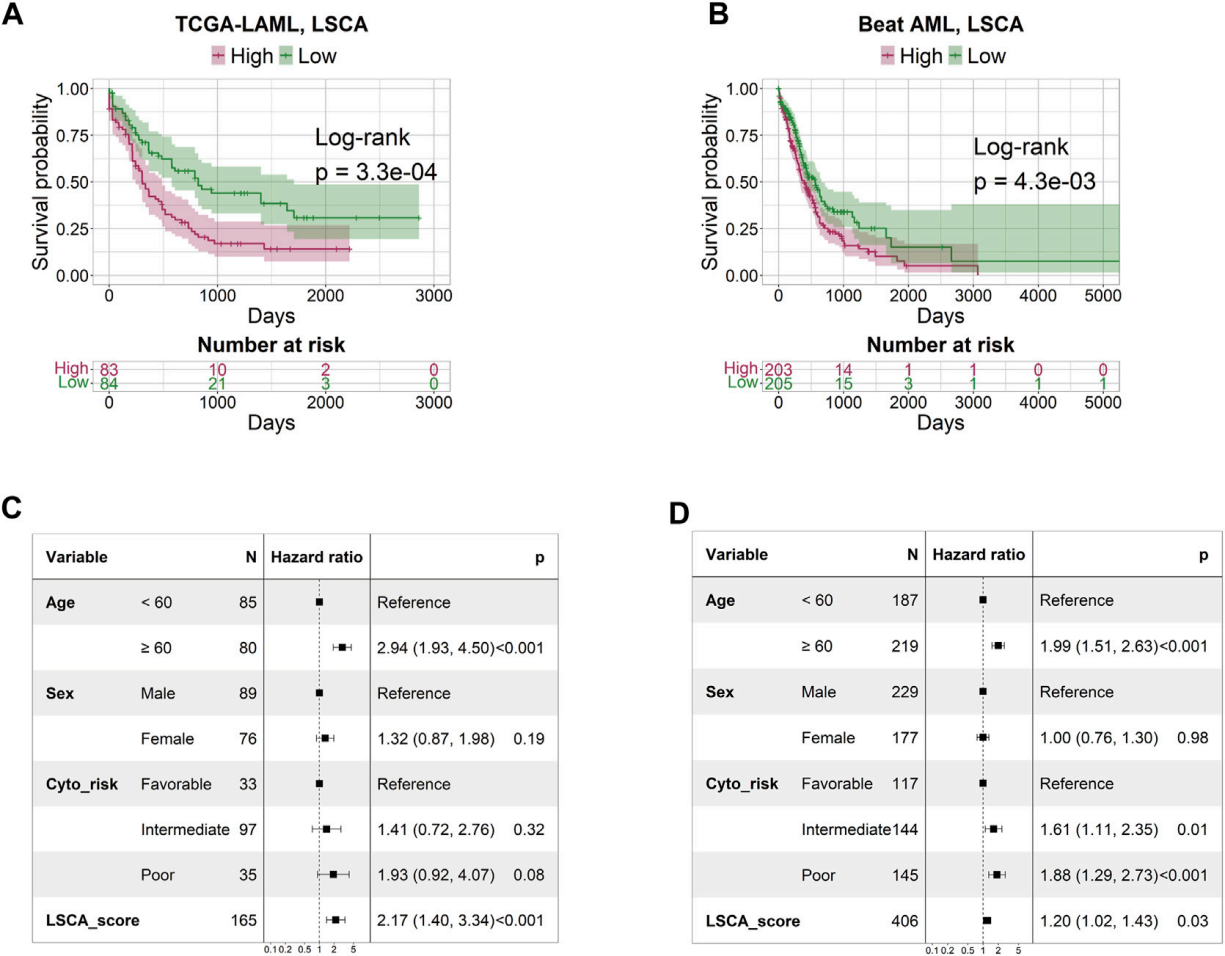
Survival and multivariate Cox regression analysis by LSCA score. The KM plot shows that the high LSCA score group tends to have a shorter survival period than the low LSCA score group in TCGA-LAML **(A)** and Beat AML **(B)** cohorts. The results of the multivariate Cox regression analysis indicate that the LSCA scores have significant hazard ratios regardless of age, sex, and cytogenetic risk in TCGA-LAML **(C)** and Beat AML **(D)** cohorts.

The patient-specific prognostic score, termed LSCA, can be calculated by summing the product of the cell type’s specific coefficient and its corresponding fraction of cell types in the sample. The LSCA scores were validated in the TCGA-LAML (*n* = 179) and Beat AML (*n* = 451) cohorts. Patients with lower LSCA scores showed favorable clinical outcomes compared to those with higher LSCA scores, the statistical significance of these was observed in both validation sets (log-rank test *p*-value = $3.3\times 10^{-4}$ and $4.3\times 10^{-3}$ , respectively) (Figures 2A, B). A multivariate Cox regression model that considered age, sex, and cytogenetic risk was used to analyze the LSCA scores as independent prognostic factors. In both data sets, HRs of the LSCA score were significantly independent of other factors (Figures 2C, D). Therefore, LSCA scores were identified as independent prognostic factors in these two data sets (HR = 2.17, 95% CI 1.40–3.34, *p* < 0.001 and HR = 1.20, 95% CI 1.02–1.43, *p* < 0.03). These findings suggest that the high LSCA score is associated with an unfavorable clinical outcome across datasets and can serve as a prognostic indicator for AML.

### Evaluation of LSCA scores

**FIGURE 3:**
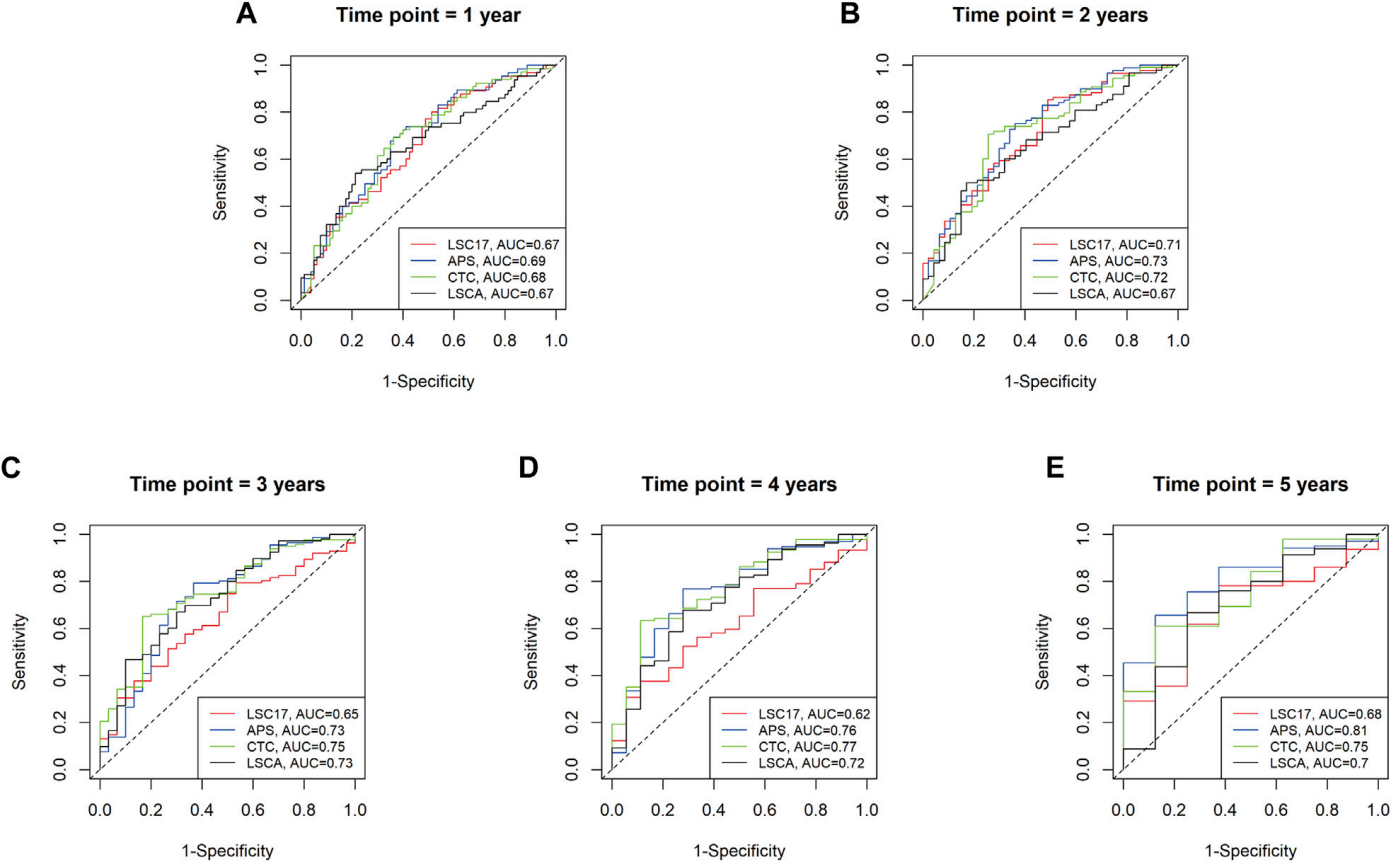
Time-dependent receiver operating characteristic (ROC) curves of four different scoring systems applied to the TCGA-LAML cohort. Area under the curves (AUCs) are similar among four different scoring systems from 1 year to 5 years **(A–E)**. No statistically significant differences between LSCA and the other three scoring systems are found at any time point (Supplementary Table S10A).

To evaluate the predictive power of the LSCA score, we compared the area under the curves (AUCs) of three leukemia-predictive scoring systems, LSC17 (Ng et al., 2016), APS (Docking et al., 2021), and CTC (Dai et al., 2021), by using a receiver operating characteristic (ROC) curves (Figure 3). We used the TCGA-LAML data sets to compare AUCs of 1, 2, 3, 4, and 5-year overall survival. In all time points examined, the LSCA score was comparable to the other three scoring systems. To verify that AUCs are not significantly different among these four scoring systems, we calculated the *p*-value using the “compare” function of timeROC R package (Blanche et al., 2013) (Supplementary Table S10A). There was no significant difference in the AUCs between the LSCA and the other three scoring systems in all time points.

Using the training and validation datasets, we compared the concordance between CTC and LSCA scores. We repeated the same strategy to calculate the CTC score of the three data sets. Concordance was evaluated for high and low-score groups using the Fisher’s exact test (FET) ( $p-\text{value}=6.7\times 10^{-6},2.2\times 10^{-8},\text{and }1.5\times 10^{-6}$ ) (Supplementary Tables S10B–D). Additionally, PCCs were calculated between CTC and LSCA scores in the three cohorts (Supplementary Figures S5A–C). Despite positive correlations between these two scores, PCCs were not larger than 0.5. It may be because these two scoring systems reflect different pathological characteristics of AML. LSCA showed better predictive power when testing on Beat AML cohort compared to CTC score (Supplementary Figures S5D–K). It is implying that predicting power of these two scoring system depends on cohort. Taken together, the performance of the LSCA and CTC scores was similar, however, they reflect different factors impacting patients’ prognosis.

### Association between LSCA score and deregulated genes in LSCs

To determine which gene sets are differentially expressed between high and low score groups, we conducted GSVA using gene expression data from the three cohorts, based on 50 hallmark gene sets (Liberzon et al., 2015) (Supplementary Figure S6). However, none of the hallmark gene set demonstrated a consistently contrasting enrichment pattern across the three cohorts between LSCA and CTC scores. Given our hypothesis that a high LSCA score better reflects the activity of LSCs compared to the CTC score, we selected six gene sets associated with LSCs from C2 CGP gene sets from MSigDB. Gene sets such as GENTLES_LEUKEMIC_STEM_CELL_UP and GENTLES_LEUKEMIC_STEM_CELL_DN include genes expressed higher or lower in LSCs compared with leukemia progenitor cells (Gentles et al., 2010). Likewise, genes differentially expressed in LSCs compared to CD34^+^CD38^+^ cells are included in gene sets such as GAL_LEUKEMIC_STEM_CELL_UP and GAL_LEUKEMIC_STEM_CELL_DN (Gal et al., 2006). Also, the other two gene sets, EPPERT_LSC_R and EPPERT_CE_HSC_LSC, cover genes upregulated in functionally defined LSCs or both HSCs and LSCs (Eppert et al., 2011).

**FIGURE 4:**
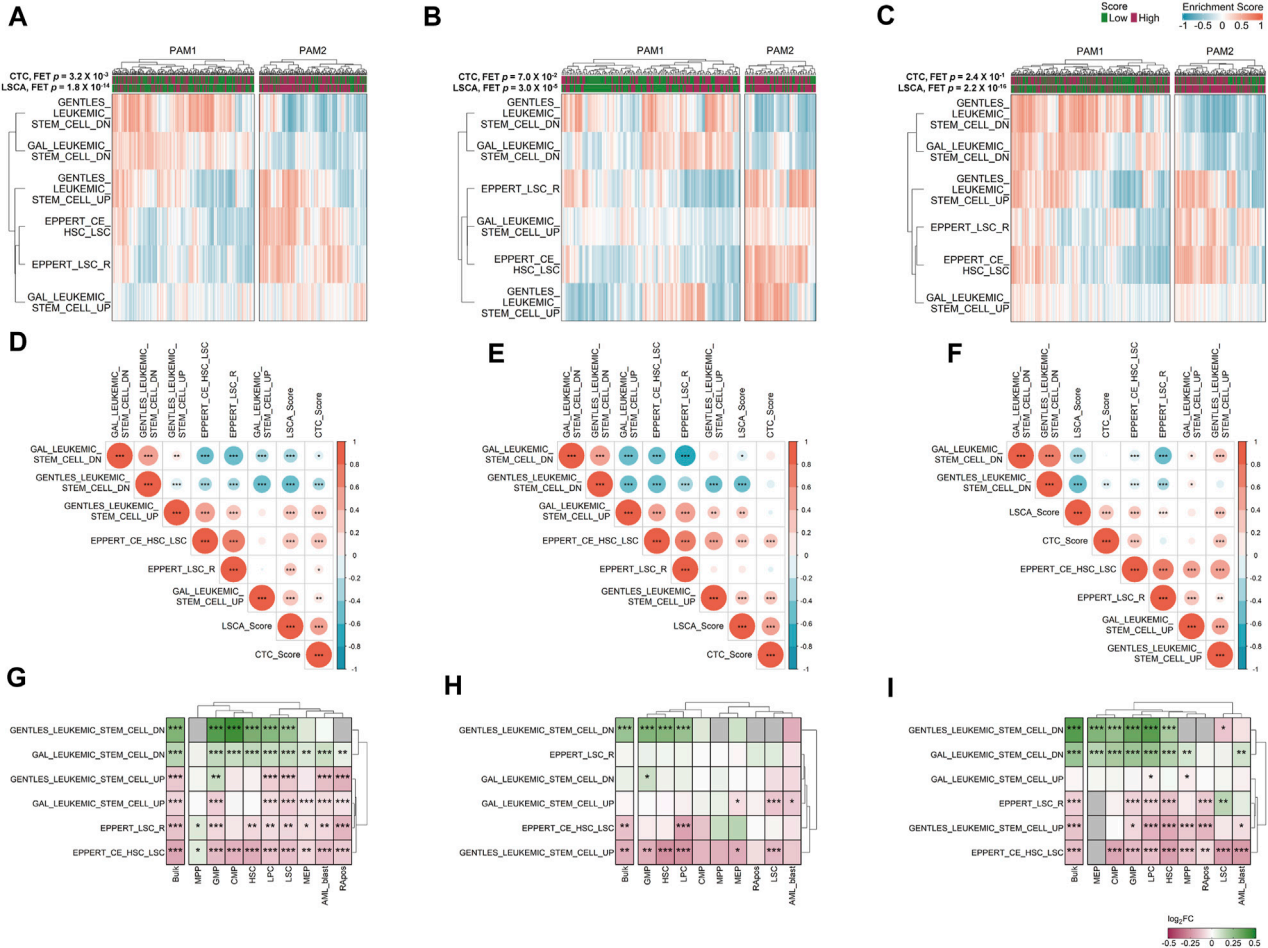
Gene set variation analysis (GSVA) in three data sets. Six manually curated LSC-associated gene sets were used to check the relationship between the LSCA score and up or downregulated genes in LSCs in GSE37642 **(A)**, TCGA-LAML **(B)**, and Beat AML **(C)** data sets. Correlation matrices shows Pearson correlation coefficients among ESs of six LSC-related gene sets and two prognosis prediction scores in GSE37642 **(D)**, TCGA-LAML **(E)**, and Beat AML **(F)** data sets. Asterisks represent the significance of the adjusted *p*-value, *, **, and *** mean less than or equal to 0.05, 0.01, and 0.001, respectively. ES log 2-fold change values of six LSC-related gene sets between high and low LSCA score groups were calculated in GSE37642 **(G)**, TCGA-LAML **(H)**, and Beat AML **(I)**. The adjusted *p*-values are tagged by asterisks in the same manner of **(D–F)**. Gray cells represent cases where there are no valid genes in the gene sets. Red represents high enrichment of the gene set in LSCA score high group, and green means high enrichment of the gene set in LSCA score low group.

We clustered samples of three cohorts by partitioning around medoids (PAM) with ES calculated by GSVA, and the two subgroups were named PAM1 and PAM2 (Figures 4A–C). Overall, upregulated genes in LSCs were enriched in PAM2 whereas downregulated genes were enriched in PAM1. In addition, patients included in the high-score LSCA group were more often found in the PAM2 group of the two validation sets (FET *p*-value = $3.0\times 10^{-5}$ and $2.2\times 10^{-16}$ ). On the other hand, the patients with a high CTC score were not significantly enriched in PAM1 or PAM2 of the two validation sets (FET *p*-value = $7.0\times 10^{-2}$ and $2.4\times 10^{-1}$ ). This implies that a high LSCA score can reflect upregulated genes in LSC better than the CTC score. In the training set, similar results were obtained although FET *p*-values were significant not only in the LSCA but also in the CTC score (Figure 4A).

We can merge LSCA and CTC scores into an ES matrix composed of gene set rows and patient columns. With this merged matrix we can calculate the PCCs among gene sets and scores (Figures 4D–F). Overall, in all three cohorts, two scores showed positive correlations with upregulated gene sets in LSCs. In contrast, the scores showed negative correlations with downregulated gene sets in LSCs. This tendency was more clearly shown in the LSCA than in the CTC score. Additionally, four upregulated or two downregulated gene sets in LSCs showed mutually exclusive positive correlations.

### GSVA of cell type-specific gene expression data

We investigated whether six LSC-related gene sets are differentially expressed between the high and low LSCA score groups not only in bulk but also in cell type-specific gene expression data. Although we can acquire sample-specific gene expression values by cell type using CIBERSORTx high-resolution mode, there are too many invalid genes which have the same expression value or NAs in every sample (Supplementary Figure S7). Nonetheless, as in the bulk expression data, the high LSCA score group showed a higher expression of upregulated genes in LSCs in cell type-specific expression data and *vice versa* (Figures 4G–I). However, the MPP and GMP of GSE37642 and LSC of Beat AML showed opposite tendency in gene sets composed of relatively small genes, such as GENTLES_LEUKEMIC_STEM_CELL_DN/UP (*n* = 19 and 29), EPPERT_LSC_R (*n* = 41), and EPPERT_CE_HSC_LSC (*n* = 42). It may be because the number of genes which have valid expression values is too low in these cell types.

## Discussion

In this study, we present a new scoring system called the LSCA score predicting the prognosis of patients based on nine cell-type fractions. In both training and validation sets, we found that the high LSCA score has an adverse impact on the prognosis and survival of patients. The inference of cell type abundances by deconvolution tool such as CIBERSORTx is primarily dependent on gene expression data of multiple genes as signature for robust determination of cell type abundances. While scoring systems like LSC17 or APS rely on a smaller, but clinically relevant set of 17 or 16 genes, the signature matrices for CTC and LSCA scoring incorporate several hundred of genes for robust estimation of cellular abundance. This difference presents a trade-off between the clinical practicability, which favors fewer genes, and the robustness achieved by using a large number of gene.

Although it may appear contradictory that LSC abundance is not factored into the LSCA score equation, the model still reflects the relationship between the LSC and AML patient prognosis. This is because patients with high LSCA scores tend to show overexpression of genes known to be upregulated in LSCs (Figure 4). In our model, instead of LSCs, MPPs showed the largest positive coefficient value. Moreover, six MPPs DEGs (*HLF*, *SETBP1*, *HOPX*, *RBPMS*, *SLC37A3*, and *TMEM200A*) are included in the LSC signature compiled by Gentles et al. (2010) (Supplementary Table S1; Supplementary Figure S1F). In summary, our LSCA scoring system is able to quantify the impact of LSCs’ activity on a patient’s prognosis, which might not directly correlate with LSCs’ abundance.

Since AML is characterized by the immature differentiation of myeloid cells (De Kouchkovsky and Abdul-Hay, 2016), we assumed that lymphoid lineages are not associated with the pathogenesis of AML. However, in the GES signature matrix used to calculate the CTC score, lymphoid lineages such as B, T, and NK cells are covered. In contrast, the HemLin9 signature matrix contains only early progenitors and myeloid lineages, excluding lymphoid lineages. Indeed, the abundance of T cells in calculating the CTC score is an important factor together with GMP-like and HSC-like. In addition, it is worth checking similarities and differences between GMP of HemLin9 and GMP-like of GES signature matrix.

In the Beat AML data set, 16 patients are aged under 20. It is reported that pediatric AML has fewer mutations and more frequent structural variants than adult AML (Bolouri et al., 2018; Chaudhury et al., 2018). Also, another study compared gene expression by the age of patients, and many genes were differentially expressed by age (de Jonge et al., 2009). *IGKC* and *GSAP* are expressed higher in the older age group and are included in the GAL_LEUKEMIC_STEM_CELL_DN and GENTLES_LEUKEMIC_STEM_CELL_UP gene sets, respectively. Further studies may need to identify whether LSCA score can be applied to pediatric patients.

Among the 9 cell types included in HemLin9, only RApos showed a significantly different survival probability between cellular abundance high and low groups simultaneously in all three data sets (Supplementary Figure S3). RApos is a cell type derived from healthy bone marrow or umbilical cord blood and has the same cell surface marker as LSC except for CD45RA. Since this cell type has a positive coefficient in LSCA score, as its abundance increases the prognosis of patients can be affected adversely. In fact, it is reported that CD45RA can be used to identify LSC subpopulations (Kersten et al., 2016). Although RApos was obtained from healthy donors, further studies are needed to verify the associations between RApos and LSC or to investigate the probability that RApos can facilitate the proliferation of LSC.

In the training set GSE37642 (GPL96), we did multivariate Cox regression with the *RUNX1-RUNX1T1* fusion and *RUNX1* mutation status instead of cytogenetic risk, which is not available. Although it is known that patients who have *RUNX1-RUNX1T1* fusion show a better prognosis (Krauth et al., 2014), the fusion status did not show significantly high HR independently of age and LSCA score. However, the *RUNX1* mutation status showed significant HR and this result is concordant with previous studies (Greif et al., 2012) (Supplementary Figure S4).

Although the LSCA score shows comparable performance to other prognosis prediction tools, this scoring system has some limitations. First, our model is largely dependent on *in silico* calculation results of cell-type abundance by deconvolution tools such as CIBERSORTx. We demonstrated that, by using pseudo-bulk gene expression data, we can effectively estimate the abundance of different cell types through deconvolution methods. Still, because we did not use actual data by experimental technique such as scRNA-seq, *in silico* prediction may not reflect real cell type compositions in the bone marrow or the blood of patients. Second, we only considered the 9 cell types and presumed that only these cells can affect the survival of patients. The HemLin9 signature matrix includes fewer than half the number of cell types included in GES signature matrix of CTC score, which uses 21 cell types. Thus, there is a possibility that cell types critical to the determination of the survival span have been missed. In addition, as mentioned before, if lymphoid lineages are included, the prediction model may need to be changed. To achieve more accurate inferences, it is necessary to have scRNA-seq data that covers a broader range of cell types including LSCs. Lastly, as mentioned earlier, deconvolution tools such as CIBERSORTx need signature matrices, however, sometimes we cannot be certain if the signature matrices contain important genes that are expressed only in specific cell types. Furthermore, some DEGs included in HemLin9 are shared by more than 2 cell types (Supplementary Figure S1E). Thus, it is essential to validate whether the signature matrix includes genes that are known to be highly expressed in a specific cell type.

In conclusion, we have developed a scoring system called the LSCA score, which uses the LASSO Cox regression to predict the prognosis of AML patients. This score shows comparable predictive power to gene expression-based scoring systems such as LSC17 and APS and the cell type composition-based CTC score. Although we have demonstrated that a high LSCA score is associated with a poorer prognosis, further studies are needed before this scoring system can be applied clinically.

## Data Availability

The datasets presented in this study can be found in online repositories. The names of the repository/repositories and accession number(s) can be found in the article/Supplementary Material.
