# Supplementary material for: Cellular abundance-based prognostic model associated with deregulated gene expression of leukemic stem cells in acute myeloid leukemia
Source: Front Cell Dev Biol. 2024 Mar 7;12:1345660. doi: 10.3389/fcell.2024.1345660 (PMC10958127; doi:10.3389/fcell.2024.1345660)
Supplement: Supplementary file 2 [file Image1.pdf]

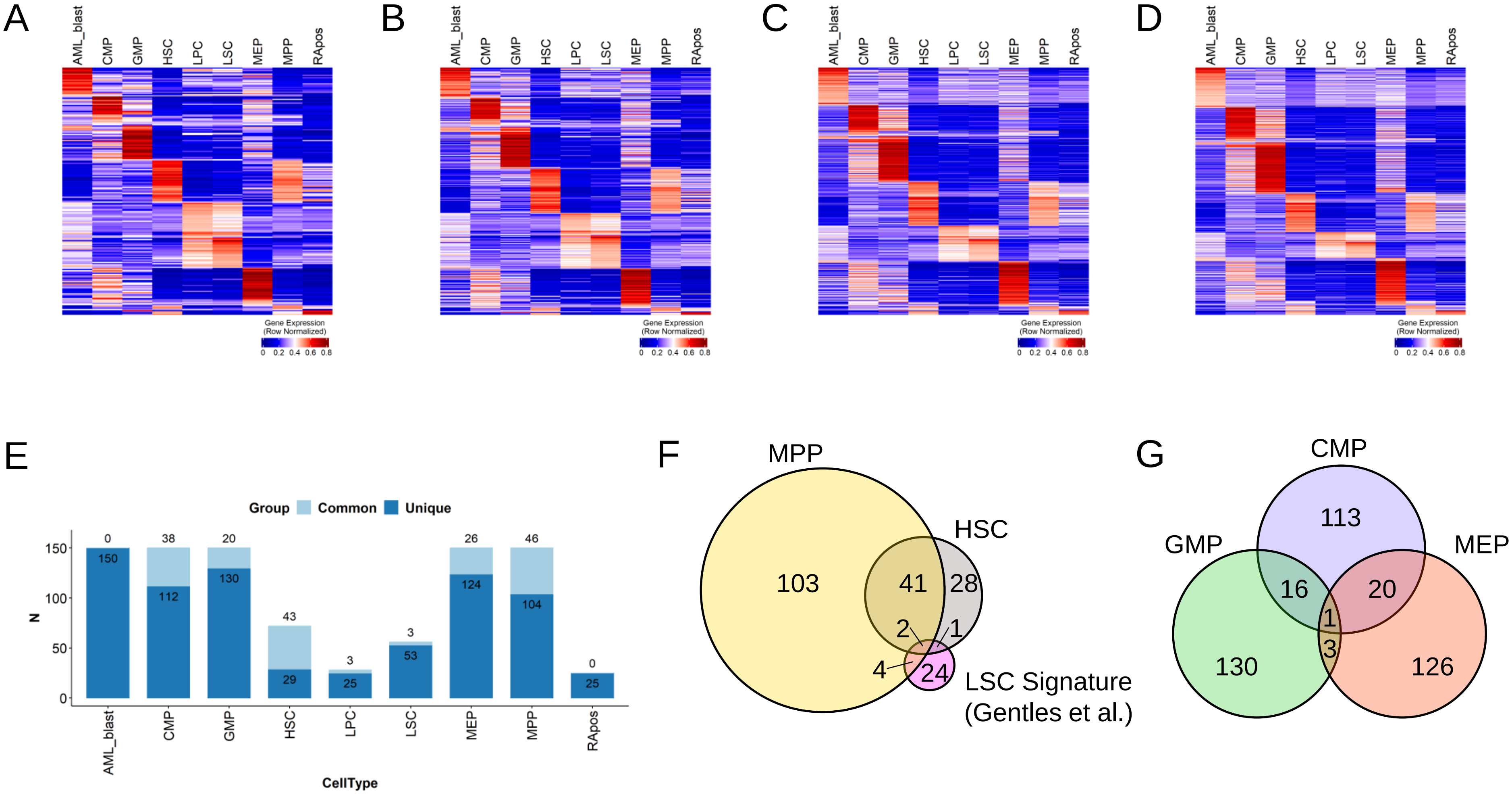

Fig. S1. Cell Type-Specific DEGs Matrices and shared DEGs. DEGs matrices composed of nine different cell types are depicted as heat maps. Each matrix is composed of 25, 50, 100, or 150 cell type-specific DEGs and depicted in **(A)**, **(B)**, **(C)**, and **(D)** respectively. DEGs counts by cell type are shown in a bar plot **(E)**. DEGs belonging to only one cell type are tagged as "Unique" and DEGs belonging to more than one cell type are represented by "Common". **(F)** MPP shares 43 DEGs with HSC, and it has six DEGs in common with the LSC signature genes (Gentles et al.). **(G)** CMP shares 17 and 21 DEGs with GMP and MEP, respectively.

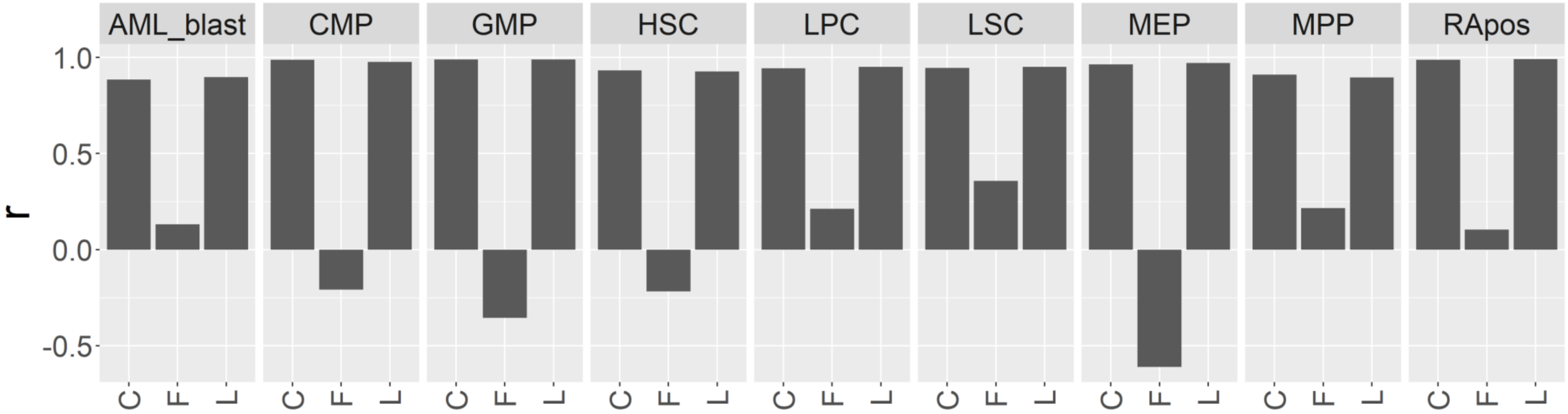

Fig. S2. Performance comparison of three cell type abundance deconvolution tools. Pearson correlation coefficients were calculated with pseudo-bulk gene expression data composed of 100 samples. Pseudo-bulk gene expression data were created by multiplying the medians of gene expression values of the same cell type and random cell type fractions using cell type sorted gene expression data (GSE24006). Then, expression values of nine cell types were summed for each sample. Cell type abundances were calculated by these three tools using HemLin9 and pseudo-bulk expression data. The correlation coefficients were calculated between the random cell type abundances and the inferred abundance values by three different tools (C – CIBERSORTx, F – FARDEEP, and L – LinDeconSeq).

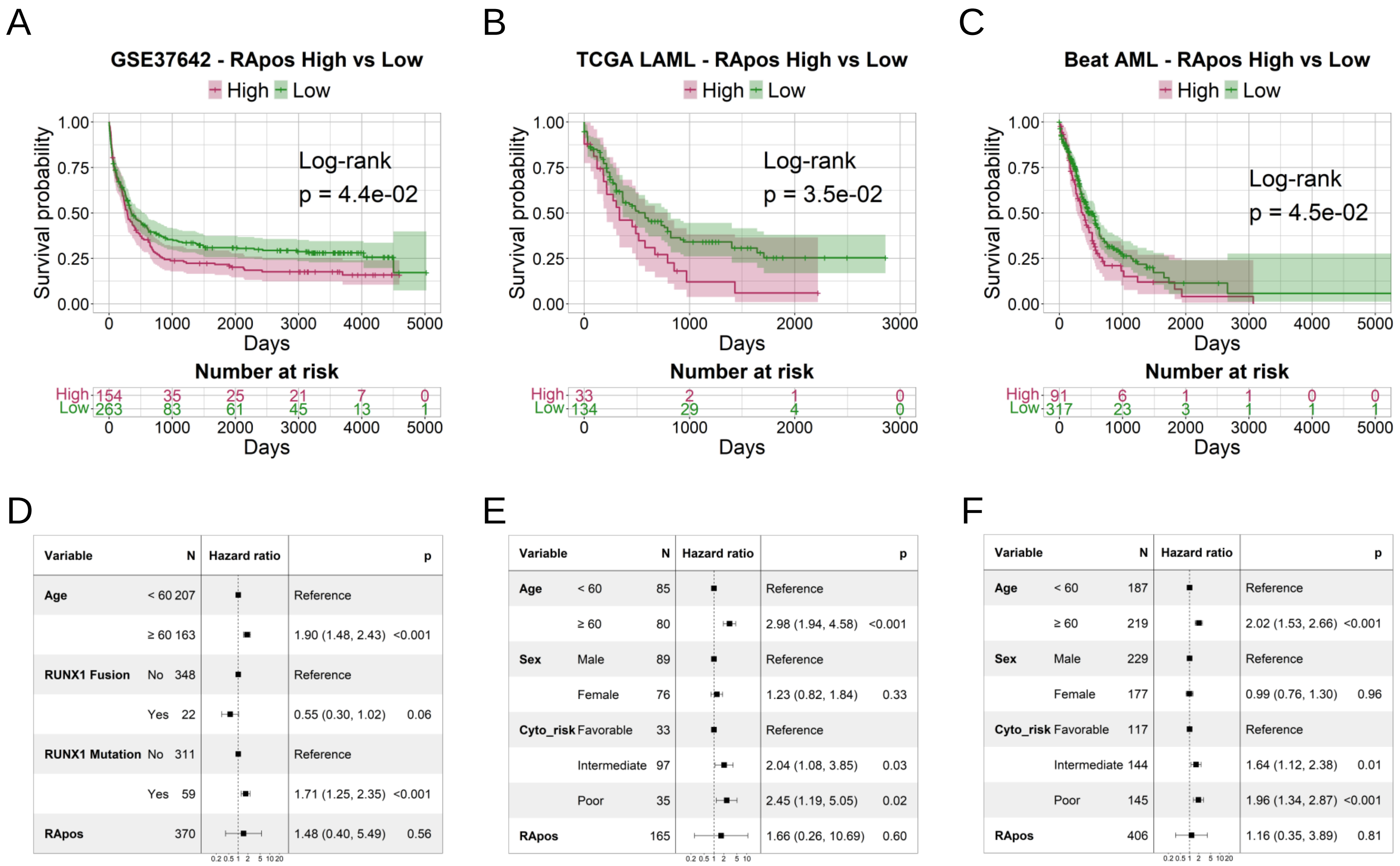

Fig. S3. Survival analysis and forest plot by RApos abundance. (A)-(C) KM plots are shown, indicating significantly different survival probabilities between RApos high and low groups. High and low groups are divided by the mean value of RApos fractions of each dataset. **(D)-(F)** Multivariate Cox regression results are shown as forest plots. Hazard ratio *p*-values of RApos abundance are not statistically significant in all three datasets. “RUNX1 Fusion” refers to cases with fusion genes of RUNX1 and RUNX1T1.

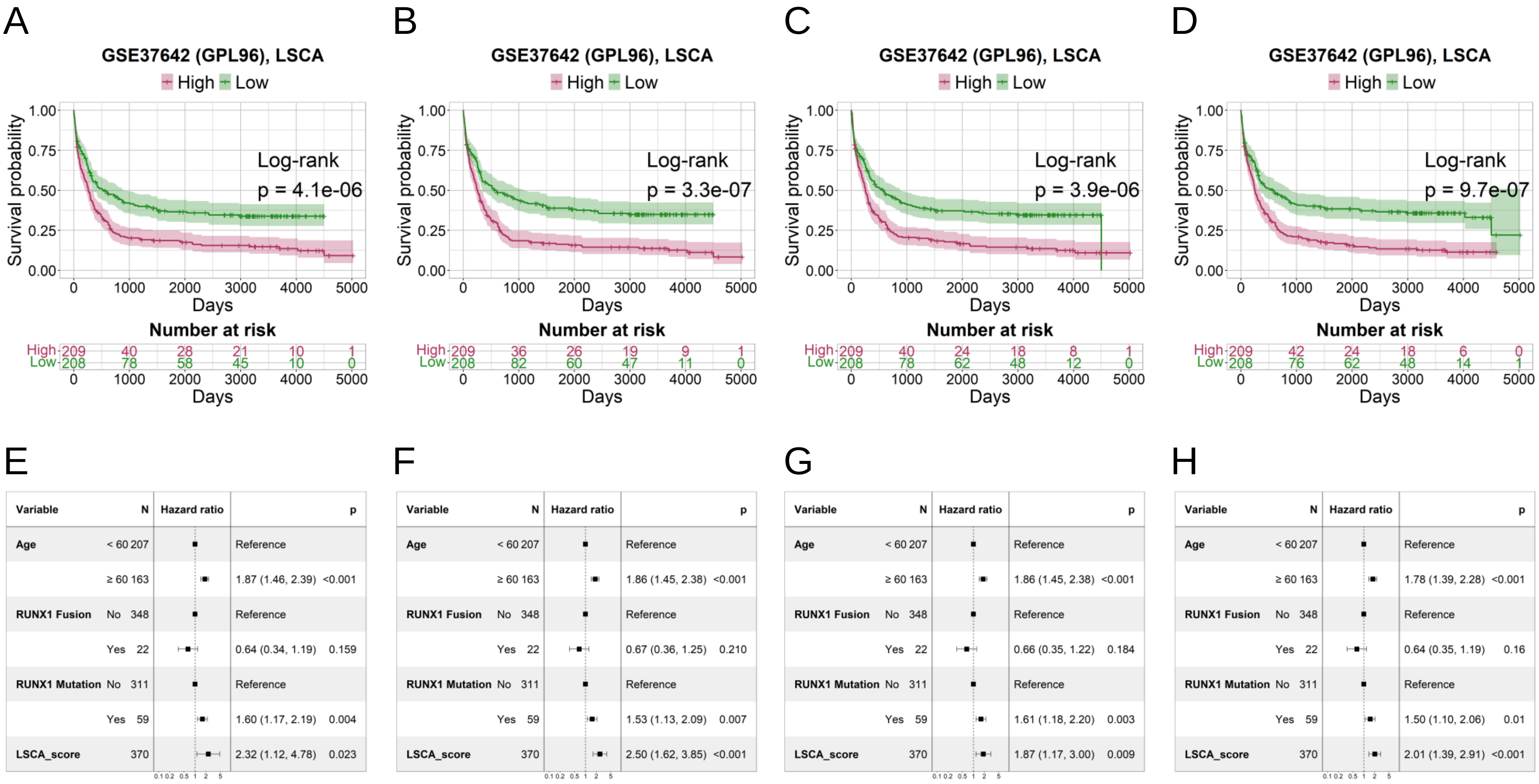

Fig. S4. Prognosis of patients based on LSCA score in the training set. Survival analysis results for the training set between the high and low LSCA groups are presented when using 25, 50, 100, and 150 DEGs (A)-(D). Multivariate Cox regression results for GSE37642 (GPL96) using 25, 50, 100, and 150 DEGs are displayed as a forest plot (E)-(H). "RUNX1 Fusion" denotes cases with fusion genes of RUNX1 and RUNX1T1.

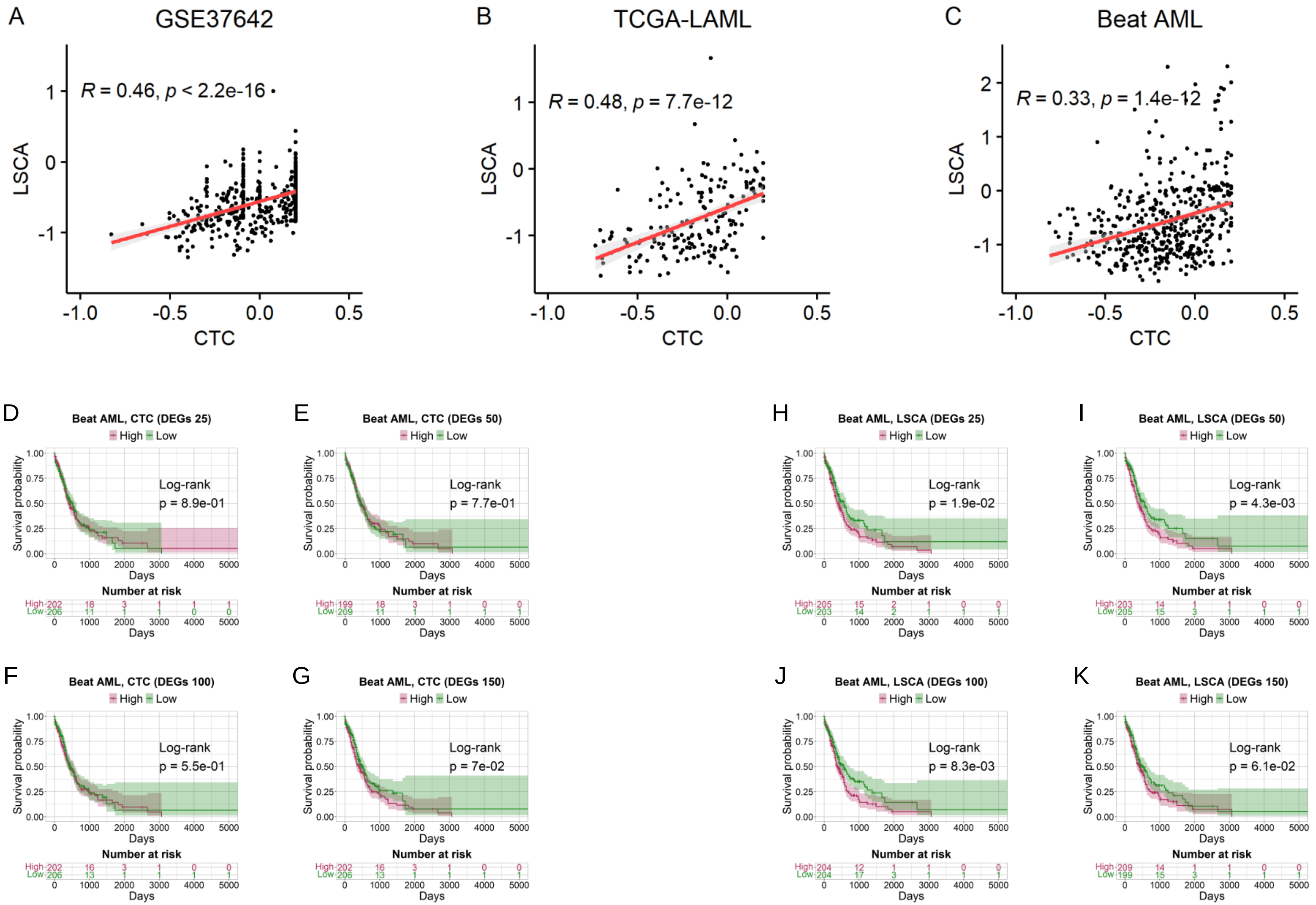

Fig. S5. Comparison of CTC and LSCA Scores by correlation and survival analysis. Pearson correlation coefficients (R) between CTC and LSCA scores were calculated in GSE37642 **(A)**, TCGA-LAML **(B)**, and Beat AML cohorts **(C)**. Survival analysis was conducted on Beat AML cohorts, comparing the high and low score groups separated by CTC **(D)-(G)** and LSCA scores **(H)-(K)**. Four signature matrices for each score were created using 25, 50, 100, and 150 DEGs. High and low groups were determined based on the median scores for each test.

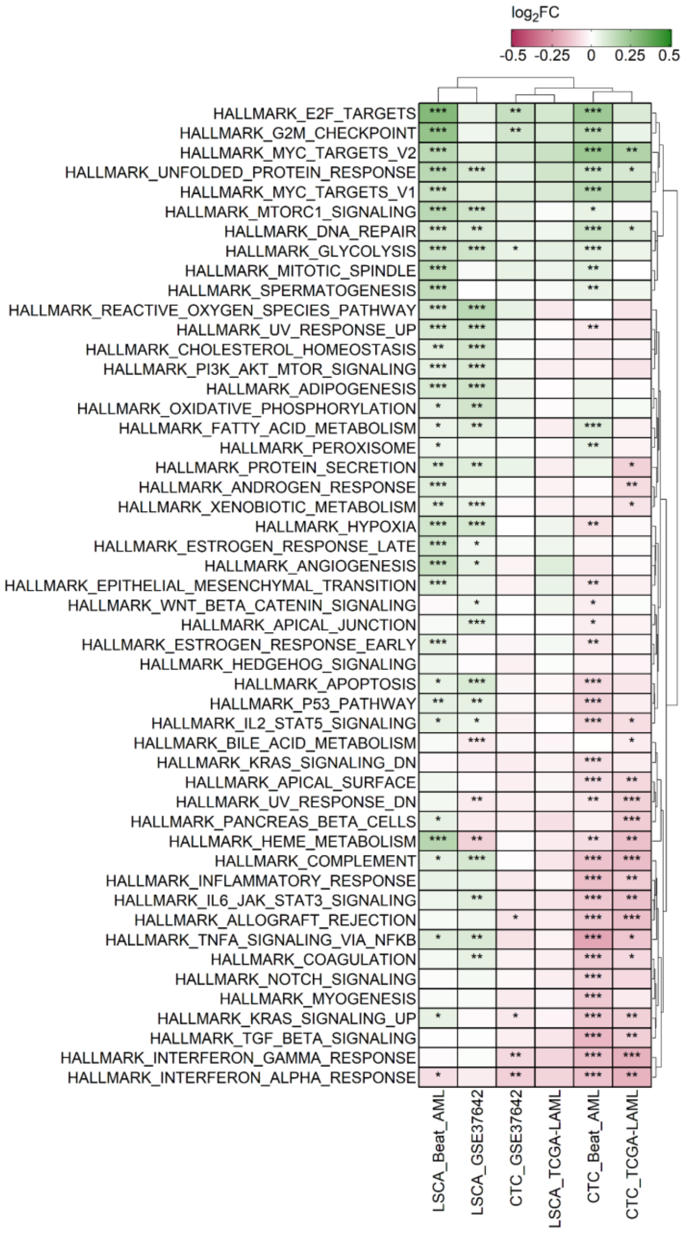

Fig. S6. Gene set variation analysis (GSVA) with hallmark gene sets between LSCA or CTC score high and low groups. Green indicates high enrichment of the gene set in the low score group, while red indicates high enrichment of the gene set in the high score group. Asterisks denote the significance of the adjusted p-values (*, **, and *** denote adjusted p-value less than or equal to 0.05, 0.01, and 0.001, respectively).

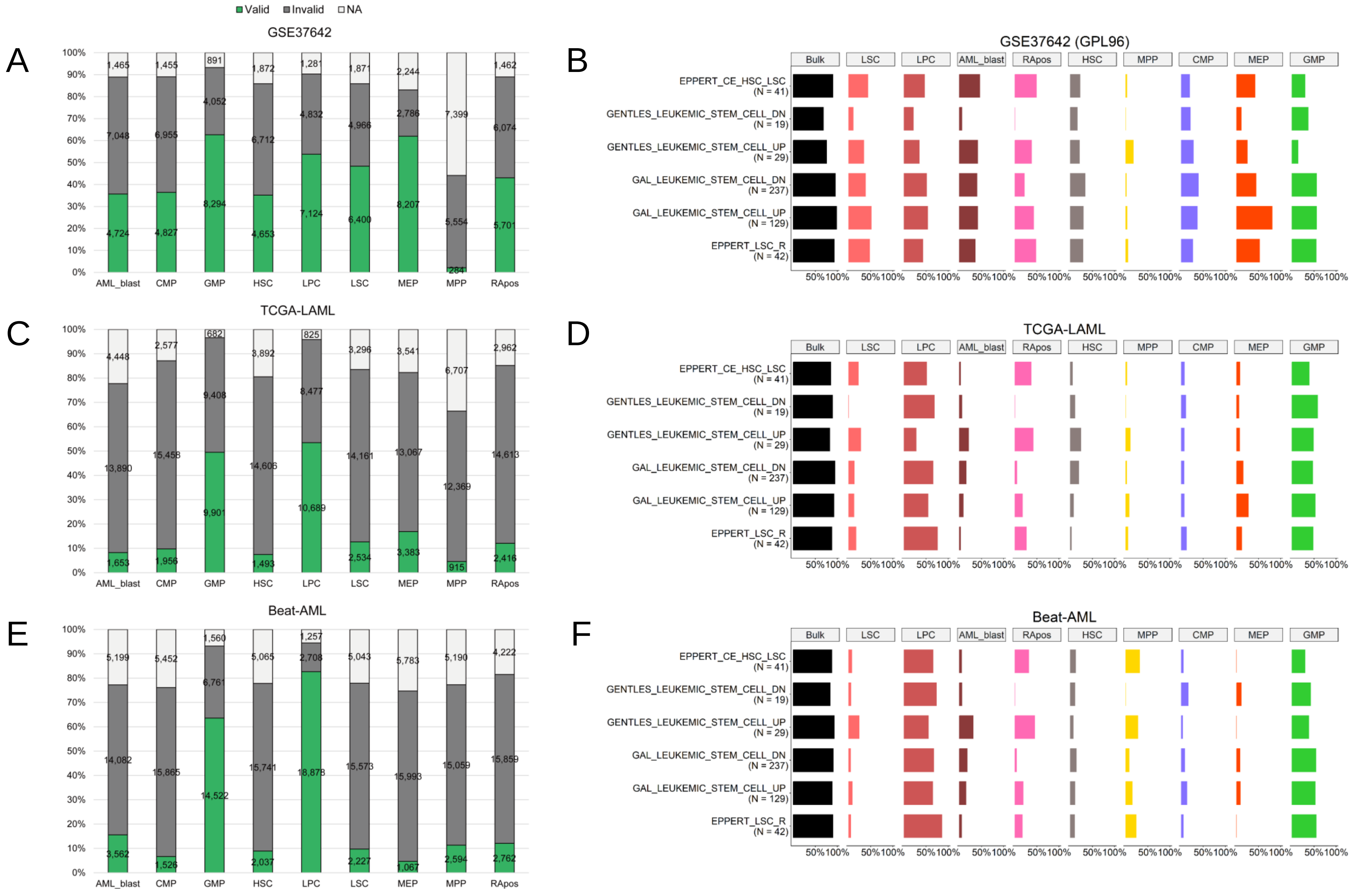

Fig. S7. Percentage of valid genes by gene set and cell type. Bar plots depict the proportion of valid genes based on CIBERSORTx high-resolution analysis. Genes with the same expression value or NA in every sample are labeled as "Invalid" or "NA," respectively. The remaining valid genes are represented in green in GSE37642 **(A)**, TCGA-LAML **(C)**, and Beat AML **(E)**. The proportion of valid genes in LSC-related six gene sets is illustrated as a bar plot by cell type in GSE37642 **(B)**, TCGA-LAML **(D)**, and Beat AML **(F)**.
